# D-dimer can be a diagnostic marker for cisplatin-related aortic thrombosis: A case report

**DOI:** 10.1097/MD.0000000000024695

**Published:** 2021-02-19

**Authors:** Yu Matsumoto, Yasushi Horimasu, Kakuhiro Yamaguchi, Shinjiro Sakamoto, Takeshi Masuda, Taku Nakashima, Shintaro Miyamoto, Hiroshi Iwamoto, Kazunori Fujitaka, Hironobu Hamada, Noboru Hattori

**Affiliations:** Department of Respiratory Medicine, Hiroshima University Hospital, 1-2-3 Kasumi, Minami-ku, Hiroshima, Japan.

**Keywords:** acute aortic thrombosis, cisplatin, D-dimer

## Abstract

**Rationale:**

Cisplatin is one of the key drugs that is frequently used for treating various types of malignancies. Although renal and digestive toxicities are well-known cisplatin-related toxicities, attention should also be paid to acute aortic thrombosis, a relatively rare but potentially fatal disorder caused by cisplatin. Additionally, D-dimer is mainly measured to detect venous thromboembolism or disseminated intravascular coagulation, whereas its usefulness for detecting aortic thrombosis remains unclear. Here, we report a case of squamous cell lung cancer treated with cisplatin-based chemotherapy, wherein acute aortic thrombosis was diagnosed based on elevated D-dimer levels.

**Patient concerns:**

A 65-year-old man with stage IV squamous cell lung cancer presented with elevated D-dimer levels during treatment with second-line chemotherapy with cisplatin and S-1. Contrast-enhanced computed tomography (CT) revealed an intramural thrombus, which had not been previously identified, extending from the abdominal aorta to the common iliac artery.

**Diagnoses:**

We diagnosed the patient as having acute aortic thrombosis caused by cisplatin.

**Interventions:**

The patient received intravenous administration of unfractionated heparin for 9 days followed by oral warfarin.

**Outcomes:**

One month after initiating treatment, the patient's D-dimer levels decreased to the normal range, and contrast-enhanced CT revealed that the thrombi had nearly completely disappeared without any sequelae or organ damage.

**Lessons:**

Our findings revealed that cisplatin can cause acute aortic thrombosis and that regular measurements of D-dimer levels before and during chemotherapy may contribute to the early detection of acute aortic thrombosis.

## 1 Introduction

Cisplatin is one of the most commonly used drugs in the treatment of various types of malignancies, including lung cancer. Renal and digestive toxicities are well-known cisplatin-related toxicities, and it has been reported that 18.1% of cisplatin-treated cancer patients develop thromboembolic events (TEEs).^[1]^ However, most of these TEEs are venous thrombosis, and cisplatin-induced acute aortic thrombosis is a rare and adverse event.^[1]^ Nonetheless, it is clinically important to promptly and accurately detect aortic thrombosis because it may cause a fatal ischemic event.

D-dimer, a marker of fibrin degradation, is mainly measured to detect venous thromboembolism or to diagnose and monitor disseminated intravascular coagulation. However, the efficacy of measuring D-dimer levels to detect aortic thrombosis remains unclear.^[2]^ Herein, we report a case of squamous cell lung cancer treated with cisplatin-based chemotherapy, wherein elevated D-dimer levels led us to the diagnosis of acute aortic thrombosis.

## 2 Case report

A 62-year-old man with a 75 pack-year smoking history was diagnosed with squamous cell lung carcinoma in June 2018. A chest computed tomography (CT) scan revealed a 42 × 50-mm-sized lesion in the upper lobe of the left lung, and whole-body 18F-fluorodeoxyglucose-positron emission tomography (FDG-PET) revealed metastasis in the right iliac bone. Following the clinical stage assessment (stage IVA [cT3N1M1b]), the patient underwent first-line chemotherapy with pembrolizumab. The treatment was discontinued after administering 4 cycles of pembrolizumab, even though its efficacy was determined as partial response. This was because the patient developed oral mucosal disorder and hypothyroidism that were considered to be immune-related adverse events caused by pembrolizumab. In May 2019, after the patient remained in remission for over 8 months, disease progression was detected using FDG-PET, which showed an abnormal uptake in the left lung tumor, left hilar lymph node, and left adrenal gland.

The patient was in a good condition with an Eastern Cooperative Oncology Group performance status of 0. He had no medical history of TEE, however, he had dyslipidemia and hyperuricemia. He had no family history of thromboembolic disease, and physical examination revealed no abnormal findings. Blood test results showed no abnormalities, including normal D-dimer levels (0.6 μg/mL). Echocardiography showed normal left ventricular systolic function without any mural thrombus.

**Figure 1:**
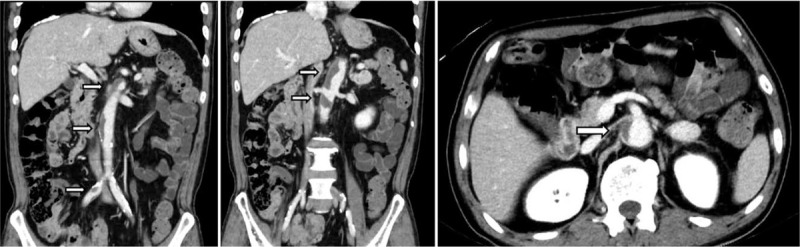
Contrast-enhanced CT on day 21: thrombotic deposits (arrows) were identified in the abdominal aorta and common iliac artery without any occlusive lesion or progression of cancer. CT = computed tomography.

**Figure 2:**
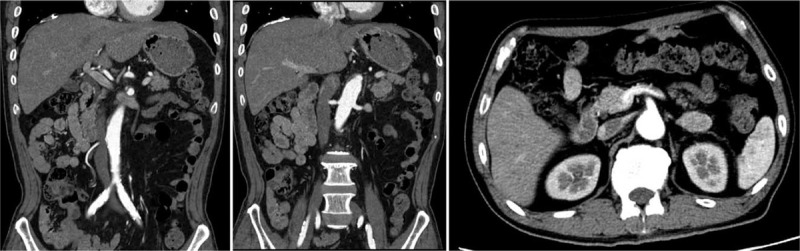
Contrast-enhanced CT on day 44: thrombus nearly completely disappeared. CT = computed tomography.

The patient was then treated with second-line chemotherapy comprising 60 mg/m^2^ cisplatin intravenously, which was administered on day 8, and 100 mg S-1 was orally administered daily from days 1 to 21. On day 14, the patient collapsed due to syncope after defecation, however, he recovered quickly without any sequelae. The patient was diagnosed with orthostatic hypotension based on a positive head-up tilt test; however, no abnormal findings, besides elevated D-dimer levels up to 8.1 μg/mL, were noted on electrocardiography or blood test results. On day 21, although no significant clinical symptoms were noted, contrast-enhanced CT performed to identify the causative lesion of elevated D-dimer levels revealed an intramural thrombus extending from the abdominal aorta to the common iliac artery without any occlusive lesion or progression of cancer (Fig. 1). Based on the patient's clinical course, he was diagnosed as having acute aortic thrombosis caused by cisplatin. To prevent further complications, he immediately received 10,000 to 20,000 U/d unfractionated heparin intravenously for 9 days and 3 mg warfarin orally. The D-dimer levels normalized on day 34, and follow-up contrast-enhanced CT performed on day 44 revealed that the thrombi in the abdominal aorta and common iliac artery had nearly completely disappeared (Fig. 2). Following this, he has been treated with chemotherapy comprising various anti-cancer agents, except for cisplatin, and no recurrence of thrombosis has been identified ever since.

## 3 Discussion

We report the case of a man with squamous cell lung cancer who developed acute aortic thrombosis during chemotherapy with cisplatin. Although thrombosis-related symptoms were not remarkable, elevated D-dimer levels led us to perform contrast-enhanced CT, which in turn led to the diagnosis of aortic thrombosis. We consider 2 clinical observations to be crucial in this case report: first, cisplatin-based chemotherapy can cause acute aortic thrombosis, and second, regular measurements of D-dimer levels before and during chemotherapy may contribute to the early detection of acute aortic thrombosis.

Cisplatin-based chemotherapy can cause acute aortic thrombosis. Moore et al^[1]^ reported that among 932 patients treated with cisplatin, 169 (18.1%) experienced TEEs during treatment or within 4 weeks after the last dose. Among the 169 patients, 150 (88.8%) had deep venous thrombosis and/or pulmonary embolism, whereas only 19 (11.2%) patients had arterial thrombosis including acute aortic thrombosis.^[1]^ In the present case, we observed the aortic thrombus without any other causative factors including occlusive lesion or progression of cancer. Therefore, we diagnosed him as having acute aortic thrombosis caused by cisplatin.

Acute aortic thrombosis, which occurred during cisplatin-based chemotherapy, has previously been reported in 13 cases (9 with lung cancer,^[3–8]^ 2 with gastrointestinal cancer,^[3,9]^ 1 with testicular seminoma,^[10]^ and 1 with cervical cancer^[11]^) (Table 1).^[11]^ Eight of the 13 previous cases, as well as our case, presented with thrombi in the abdominal aorta, whereas in 4 cases, thrombi were limited to the thoracic aorta. In previous cases, thrombi were detected between 12 days and 5 months after initiating cisplatin-based chemotherapy. In our case, we identified thrombi on a CT scan on day 21, which is relatively early in the treatment course as compared with the average of the previous cases. Furthermore, etoposide or vinorelbine was used in combination with cisplatin in most of the previous cases, whereas S-1 was administered to only 1 patient besides the one in our case report.

**Table 1:** Reported cases of cisplatin-related aortic thrombosis.

Case	Author	Age/sex	Cancer type	Chemotherapy	Location of thrombi	D-dimer	Detection opportunity	Detection time	Management
1	Hahn et al^[5]^	74/M	Lung cancer (adenocarcinoma)	CDDP/VP16	Ascending aorta	1077 μg/L (0–324 μg/L)	Symptom (dyspnea on exertion and chest discomfort)	N/A	Anticoagulant
2	Ito et al^[9]^	66/F	Gastric cancer	CDDP/S-1	Descending arch of the thoracic aorta	(FDP: 3.4 μg/mL)	Accidental (follow-up CT)	Follow up after the first cycle of chemotherapy	Anticoagulant
3	Hahn et al^[5]^	50/M	Lung cancer (small cell carcinoma)	CDDP/VP16	Aortic arch	3.2 μg/mL	Accidental (follow-up CT)	Approximately 6 weeks after cisplatin was first administered	Anticoagulant
4	Dieckmann et al^[10]^	49/M	Testicular seminoma	CDDP/VP16/BLM	Descending arch of the thoracic aorta and infrarenal abdominal aorta	N/A	Accidental (follow-up CT)	Follow up after the second cycle of chemotherapy	Anticoagulant
5	Fernandes et al^[3]^	60/F	Rectosigmoid adenocarcinoma	CDDP/5-FU/FLO	Proximal abdominal aorta and extending to the right common iliac artery	N/A	Accidental (follow-up CT)	Six days after completion of the 3rd cycle	Anticoagulant
6	Fernandes et al^[3]^	53/M	Lung cancer (small-cell lung adenocarcinoma)	CDDP/VP16	Abdominal aorta, extending from the level of the celiac artery to the right common iliac artery	N/A	Symptom (vomiting and abdominal pain)	Approximately 3 months after cisplatin was first administered	Anticoagulant
7	Fernandes et al^[3]^	53/M	Lung cancer (adenocarcinoma)	CDDP/VNR	Abdominal aorta, extending from the level of the celiac plexus to the left common iliac artery	N/A	Accidental (follow-up CT)	Approximately 4.5 months after cisplatin was first administered	Anticoagulant
8	Fernandes et al^[3]^	50/F	Non-small cell lung cancer	CDDP/VNR	Abdominal aorta, extending from the level of the superior mesenteric artery to the level above the origin of the common iliac arteries.	N/A	Accidental (follow-up CT)	Approximately 4.5 months after cisplatin was first administered	Anticoagulant
9	Sato et al^[6]^	68/M	Lung cancer (adenocarcinoma)	CDDP/VNR	From the thoracic artery to the abdominal aorta	5.7 μg/mL	Symptom (acute pain in his right leg and intermittent claudication)	Thirteen days after cisplatin was first administered	Anticoagulant
10	Mathews et al^[7]^	54/M	Lung cancer (adenocarcinoma)	CDDP/VP16	Abdominal artery below the renal artery	N/A	Symptom (discomfort in his right leg)	Twelve days after cisplatin was first administered	Amputation (below the knee)
11	Mathews et al^[7]^	54/F	Lung cancer (large cell type)	CDDP/VP16	Abdominal artery at the level of the renal artery	N/A	Symptom (leg paresthesia and low back pain)	Halfway through the second cycle of chemotherapy	Thrombectomy aortobifemoral graft
12	Aoki et al^[8]^	64/M	Lung cancer (adenocarcinoma)	CDDP/PEM	Aortic arch	2.9 μg/mL	Accidental (follow-up CT)	Follow up after the first cycle of chemotherapy	Anticoagulant
13	Abdel-Razeq et al^[12]^	59/F	Cervical cancer	CDDP/5-FU	Aortic arch, descending aorta, and popliteal artery	N/A	Symptom (acute pain in his right leg, nausea, and vomiting)	N/A	Thrombectomy of the popliteal artery
14	Matsumoto et al (present case)	65/M	Lung cancer (squamous cell carcinoma)	CDDP/S-1	From the abdominal aorta to the common iliac artery	8.1 μg/mL	Accidental (D-dimer increase)	Thirteen days after cisplatin was first administered	Anticoagulant

Although the exact mechanism by which cisplatin causes TEE remains unknown, it has been reported that cisplatin induces damage to vascular endothelial cells via hypomagnesemia, increased activity of von Willebrand factor, and increased formation of procoagulant endothelial microparticles.^[1]^ Furthermore, platelet activation and upregulation of prothrombotic factors are implicated in cisplatin-related thrombosis.^[12]^

D-dimer is a product generated by plasmin-induced degradation of stabilized fibrin and is a generic term for mixtures having a D-dimer structure. An increase in D-dimer levels may be a marker for thrombus formation and the enhanced state of secondary fibrinolysis, and thus, is widely used in clinical settings as an important marker for thrombosis detection. As shown in Table 1, 6 of the 13 reported cases were diagnosed while investigating the source of a symptom; however, others were asymptomatic and were diagnosed based on follow-up CT, which evaluates the response to chemotherapy. These findings suggest that aortic thrombosis may follow an asymptomatic course for a period of time; however, there is a high need for its early detection because it may be exacerbated by organ embolism. In our case, D-dimer levels before initiating chemotherapy were normal and increased only after cisplatin was administered. This apparent change encouraged us to perform contrast-enhanced CT, which resulted in the diagnosis of aortic thrombosis relatively early in the treatment course compared to previous cases.

Although many studies have reported the usefulness of D-dimer in cases of intravenous thrombus and aortic aneurysm, its utility in the detection of intra-aortic thrombus remains uncertain. Nevertheless, as shown in Table 1, D-dimer levels were elevated in all 4 previous cases as well as in our case. These results suggest that D-dimer can be a potential diagnostic marker of cisplatin-related aortic thrombosis. Importantly, elevated D-dimer is commonly observed in presence of cancer no matter with or without thrombus. Therefore, regular measurements of D-dimer levels started from before the initiation of cisplatin-based chemotherapy can be useful to distinguish the elevation of D-dimer caused by the chemotherapy from that caused by the cancer itself, and it may helpful for the early detection of aortic thrombosis, especially before the appearance of thrombus-induced symptom. However, we should note that the elevation of D-dimer in patients with cancer can be caused by various factors including cancer itself or intravenous thrombus, thus the further examination such as contrast-enhanced CT is required to investigate the cause of the elevation of D-dimer.

In conclusion, we report a case of acute aortic thrombosis caused by cisplatin. Cisplatin-related arterial thrombus is a relatively rare but important complication that can cause life-threatening ischemic events. Regular measurements of D-dimer levels during chemotherapy with cisplatin may help in the early detection of acute aortic thrombosis.

## Author contributions

**Conceptualization:** Yu Matsumoto, Yasushi Horimasu.

**Data curation:** Yasushi Horimasu, Kakuhiro Yamaguchi, Shinjiro Sakamoto, Takeshi Masuda, Taku Nakashima, Shintaro Miyamoto, Hiroshi Iwamoto, Kazunori Fujitaka, Hironobu Hamada, Noboru Hattori.

**Investigation:** Yu Matsumoto, Yasushi Horimasu.

**Methodology:** Yu Matsumoto, Yasushi Horimasu.

**Supervision:** Hiroshi Iwamoto, Kazunori Fujitaka, Hironobu Hamada, Noboru Hattori.

**Writing – original draft:** Yu Matsumoto.

**Writing – review & editing:** Yu Matsumoto, Yasushi Horimasu, Kakuhiro Yamaguchi, Shinjiro Sakamoto, Takeshi Masuda, Taku Nakashima, Shintaro Miyamoto, Hiroshi Iwamoto, Kazunori Fujitaka, Hironobu Hamada, Noboru Hattori.
